# Renal vascular lesions in childhood-onset lupus nephritis

**DOI:** 10.1007/s00467-024-06498-z

**Published:** 2024-09-09

**Authors:** Kyle Ying-kit Lin, Eugene Yu-hin Chan, Yuen-fun Mak, Ming-chun To, Sze-wa Wong, Fiona Fung-yee Lai, Tsz-wai Ho, Pak-chiu Tong, Wai-ming Lai, Desmond Yat-hin Yap, Alison Lap-tak Ma

**Affiliations:** 1 Paediatric Nephrology Centre, Hong Kong Children’s Hospital, Hong Kong, Hong Kong SAR; 2 Department of Paediatrics, The Chinese University of Hong Kong, Hong Kong, Hong Kong SAR; 3 Department of Paediatrics and Adolescent Medicine, The University of Hong Kong, Hong Kong, Hong Kong SAR; 4 Department of Pathology, Princess Margaret Hospital, Hong Kong, Hong Kong SAR; 5 Division of Nephrology, Department of Medicine, Queen Mary Hospital, The University of Hong Kong, Hong Kong, Hong Kong SAR

**Keywords:** Renal vascular lesions, Childhood-onset lupus nephritis, SLE, Systemic lupus erythematosus, Non-inflammatory necrotizing vasculopathy, Thrombotic microangiopathy

## Abstract

**Background:**

This study aimed to determine the clinical significance of renal vascular lesions (RVLs) in childhood-onset lupus nephritis (cLN).

**Methods:**

We retrospectively reviewed all children with biopsy-proven cLN between 2004–2020 to evaluate the prevalence of RVLs on kidney biopsy and its associated factors and long-term outcomes. The composite kidney outcome was defined as advanced chronic kidney disease (CKD) stage 3–5, kidney failure and death.

**Results:**

107 biopsies from 84 Chinese patients were analysed. RVLs were observed in 19 patients (22.6%), including non-inflammatory necrotizing vasculopathy (NNV, *n* = 6), thrombotic microangiopathy (TMA, *n* = 4), arterial sclerosis (AS, *n* = 3), concurrent NNV with AS (*n* = 4), concurrent NNV with TMA (*n* = 1) and concurrent true renal vasculitis with AS (*n* = 1).

The presence of RVLs was associated with lower estimated glomerular filtration rate (eGFR) (66.9 ± 40.3 vs. 95.6 ± 39.4 ml/min/1.73m^2^, *p* = 0.005), haemoglobin level (9.1 ± 1.9 vs. 10.4 ± 1.9 g/dL, *p* = 0.008) and platelet count (150.1 ± 96.4 vs. 217.2 ± 104.8 × 10^9^/L, *p* = 0.01). LN classes and activity/chronicity indices were similar.

Patients with RVLs had poorer composite kidney outcomes, though not reaching statistical significance (log-rank test, *p* = 0.06). The presence of NNV was associated with inferior survival free from composite kidney outcome (log-rank test, *p* = 0.0018), compared to other forms of RVLs and those without RVLs. Univariate analysis revealed NNV (HR 7.08, 95% CI 1.67–30.03) was predictive of composite kidney outcome.

**Conclusion:**

RVLs are present in one-fifth of cLN patients and are associated with severe presentation. NNV is associated with worse long-term kidney outcome. Routine evaluation of RVLs is warranted and should be incorporated into future classification criteria.

**Graphical Abstract:**

A higher resolution version of the Graphical abstract is available as Supplementary information

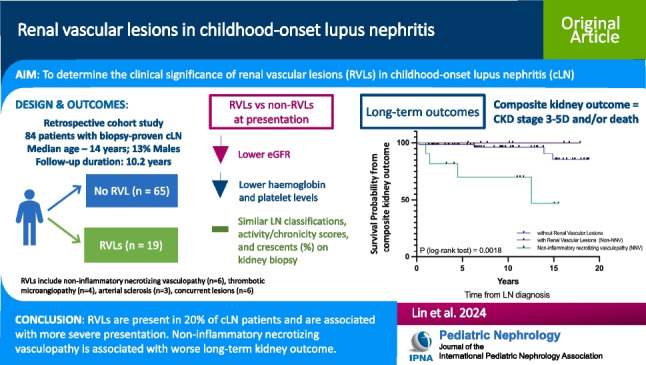

**Supplementary Information:**

The online version contains supplementary material available at 10.1007/s00467-024-06498-z.

## Introduction

Kidney involvement is common in childhood-onset systemic lupus erythematosus (cSLE), occurring in around 50–80% of patients [1, 2]. Around 4% of cSLE develop advanced chronic kidney disease (CKD) [2, 3]. Up to 15% of patients with childhood-onset lupus nephritis (cLN) may progress to kidney failure [4–6]. It is also associated with significant morbidity and mortality, compared to the general paediatric population [7].

Histopathological findings in lupus nephritis (LN) are defined according to the International Society of Nephrology/Renal Pathology Society (ISN/RPS) classification. Prompt and accurate histological evaluation is important as it guides clinical management, since pathological findings are closely correlated to disease activity, treatment response and long-term kidney outcomes [8]. Histopathological features in the ISN/RPS classification largely focus on glomerular and tubulointerstitial lesions, where renal vascular lesions (RVLs) are often overlooked. RVLs in LN include arterial sclerosis (AS), non-inflammatory necrotizing vasculopathy (NNV), thrombotic microangiopathy (TMA), true renal vasculitis (TRV) and isolated immune complex deposits. Although the current ISN/RPS 2018 classification proposes standardized terminology and definition for RVLs, these findings have not been incorporated into the classification criteria [9]. While these lesions have been reported to be associated with clinical severity and adverse kidney outcomes in adult-onset LN, their role as an independent prognostic factor remains controversial [10, 11].

cLN shows substantial differences in the disease pathogenesis, clinical presentation, epidemiology and outcomes compared to adult-onset LN [5]. There is also no currently available data pertaining to the clinical significance of RVLs in cLN. In view of these knowledge gaps, this study was done to investigate the prevalence, associated factors and long-term outcomes of RVLs in biopsy-proven cLN.

## Methods

### Design and patients

We conducted a retrospective cohort study at the Paediatric Nephrology Centre of Hong Kong Children’s Hospital, Hong Kong, which was previously located at Princess Margaret Hospital. The Paediatric Nephrology Centre is the designated referral centre for complicated childhood kidney disorders and paediatric kidney replacement therapy. All Chinese children with biopsy-proven cLN diagnosed before 18 years of age, between 1 January 2004 and 31 December 2020, were identified and included for analysis. The minimum follow-up period following initial therapy was 12 months and patients with alternative diagnoses, such as overlap syndrome, were excluded from the analysis. The diagnosis of SLE was based on 1997 American College of Rheumatology criteria and/or 2012 Systemic Lupus International Collaborating Clinics group classification criteria [12, 13]. Data retrieval and analysis was approved by the Central Institution Review Board of the Hospital Authority, Hong Kong (Reference no. PAED-2022–002).

### Kidney histopathology

Kidney biopsies were performed at the time of first kidney presentation and in selected cases with severe kidney relapses. In our centre, kidney biopsy is generally performed with indications recommended by EULAR/ERA-EDTA 2019 guidelines, i.e. glomerular haematuria and/or cellular casts, proteinuria > 0.5 g/24 h, unexplained decrease in estimated glomerular filtration rate (eGFR) [14].The kidney histology was either assessed or reviewed by the same experienced kidney pathologist during the study period, and all biopsy samples were examined with light microscopy, immunofluorescence, and most with electron microscopy. Histology was classified according to the ISN/RPS 2003 classification [8].

Five types of RVL were carefully identified and reviewed [10]: 1) AS—thickening of arterial wall and narrowing of the lumen by intimal fibrosis and/or hyaline arteriosclerosis; 2) NNV—necrotizing changes in the vessel wall associated with abundant immune complex deposits, typically admixed with fibrin, without inflammation of the vessel wall, causing significant luminal narrowing or occlusion; 3) TMA—luminal narrowing or total occlusion by intraluminal, subendothelial or medial accumulation of eosinophilic, fuchsinophilic material with staining properties of fibrin, with the association of endothelial oedema and luminal thrombi; 4) TRV—prominent inflammatory cell infiltration of the arterial wall by neutrophils and mononuclear leukocytes; and 5) Isolated immune complex deposits (ICD)—immunofluorescence study without corresponding changes on light microscopy. Isolated immune complex deposits, however, were difficult to evaluate due to retrospective assessment. Consequently, these patients with isolated immune complex deposits on immunofluorescence staining were excluded from the study.

### Therapy

All patients received standard immunosuppressive therapy for cLN (induction treatments during the first 3 to 6 months of therapy, followed by a maintenance phase). Induction therapies for patients with proliferative LN (Class III, IV and mixed III/IV + V) comprised high-dose oral prednisolone (0.8–1 mg/kg/day), with or without intravenous pulse methylprednisolone (10–30 mg/kg per dose for 3 doses, max. 1 g/dose). The other induction agents included mycophenolate mofetil (MMF, 1200 mg/m^2^/day), or in severe cases intravenous cyclophosphamide (CYC), based on National Institutes of Health (0.5–1 g/m^2^ monthly for 6 months) or Euro-Lupus regimens (500 mg every 2 weeks for 3 months). Patients with severe membranous LN (class V) were managed with a combination of oral prednisolone and MMF. The maintenance immunosuppression included low-dose prednisolone (3–5 mg/day) with either azathioprine (AZA, 1.5–2 mg/kg/day) or MMF (600–1200 mg/m^2^/day). Triple immunosuppressive therapy with corticosteroids, MMF and calcineurin inhibitors, or adjunctive treatments such as rituximab was offered to selected patients who were refractory to first-line treatments. Anti-malaria and/or renin–angiotensin–aldosterone system inhibitors were prescribed unless contraindicated. There was no specific treatment for RVLs except therapeutic plasmapheresis in the presence of severe TMA.

### Clinical evaluation

Data pertaining to patient demographics, clinical manifestations and laboratory results were obtained through electronic medical systems. Estimated glomerular filtration rate (eGFR) was calculated with serum creatinine, by revised Schwartz formula for patients younger than 18 years [15], and CKD-Epidemiology Collaboration (CKD-EPI) equation for 18-year-old patients [16]. Patients were followed every 1 to 8 weeks. Clinical parameters including blood pressure, urinalysis, physical examination findings were documented. Laboratory tests including complete blood count, kidney biochemistry, anti-dsDNA, complement C3 levels and proteinuria were monitored regularly every 1 to 8 weeks.

### Outcomes

The primary outcome was a composite kidney outcome of advanced CKD (stage 3–5, eGFR < 60 ml/min/1.73 m^2^), kidney failure requiring maintenance kidney replacement therapy (KRT) and/or mortality. Secondary outcomes included prevalence, clinical presentations, treatment response and association factors for RVLs. Complete remission (CR) was defined by KDIGO guidelines as sustained early morning urinary protein-creatinine ratio (UPCR) < 0.5 mg/mg [17]. Partial remission (PR) was defined as reduction in UPCR by ≥ 50% and < 3 mg/mg [17]. Both CR and PR required the stabilization or improvement in kidney function (± 10% ~ 15% of baseline) [17]. Kidney relapse was defined by UPCR more than 1 mg/mg in patients with baseline proteinuria less than 0.5 mg/mg or a rise in UPCR by more than 1 mg/mg in patients with baseline proteinuria more than 0.5 mg/mg and/or a rise in serum creatinine, with the support from either serological or histological evidence of disease activity [18].

### Statistical analysis

Statistical analysis was performed by IBM SPSS Statistics version 26 software. When comparing the demographics, laboratory variables, histological differences and treatment regimens, Fisher exact test or Pearson’s chi square test was used for categorical variables while Student’s t-test, Mann–Whitney U test or Kruskal–Wallis test was used for continuous variables. Survival curve and survival pattern for composite-kidney outcomes were generated by the Kaplan–Meier method. Log-rank test was used to evaluate the difference in survival probability from the composite-kidney outcomes in different groups. Univariate model was constructed with hazard ratio on individual factors with COX regression analysis.

## Results

**Fig. 1:**
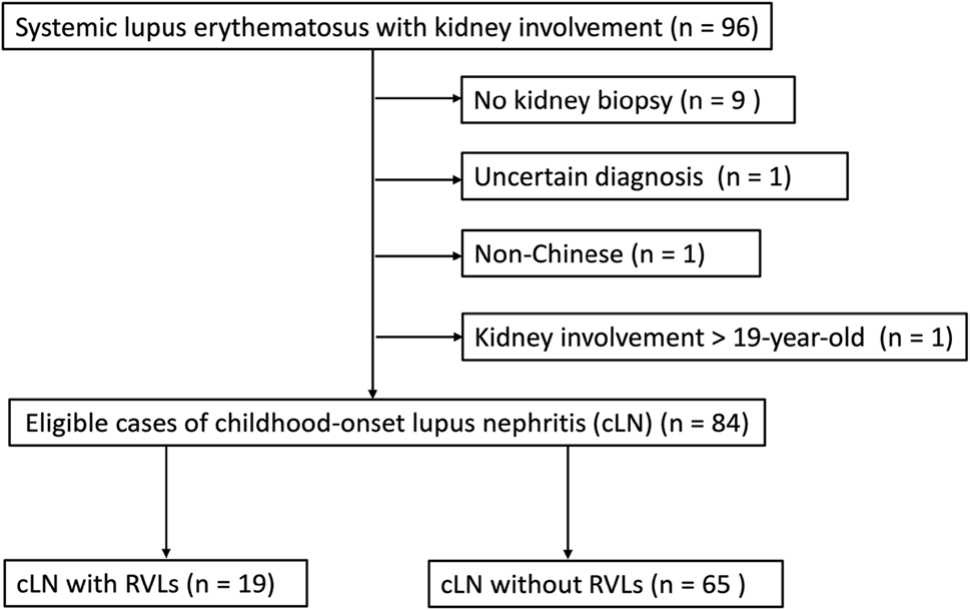
Study flow diagram, showing inclusion and exclusion of eligible cases for analysis

Ninety-six patients with cLN were identified, of which 12 patients were excluded from the study (Fig. 1). A total of 84 patients were included in the final analysis with 107 kidney biopsies performed. The median follow-up time was 10.2 years (IQR 5.7, 15.4) from LN diagnosis. RVLs were observed in biopsies of 19 patients (22.6%). Fourteen kidney biopsies with RVLs were performed at LN diagnosis, while another five were done during kidney flares. Patients with or without RVLs on kidney biopsy showed no difference in gender, age of LN diagnosis, proteinuria, anti-dsDNA, C3, SLEDAI score, and autoantibodies profile (Table 1).

**Table 1:** Baseline demographics and laboratory findings at the time of LN diagnosis in patients with and without RVLs

Clinical Characteristics	All (*n* = 84)			With renal vascular lesions (*n* = 19)			Without renal vascular lesions (*n* = 65)			*P* value
Patient demographics										
Sex, Female	73	(86.9)		16	(84.2)		59	(87.7)		NS
Age at SLE diagnosis, y	12.9	± 3.2		13.2	± 3.4		12.9	± 3.2		NS
Age at LN diagnosis, y	14.0 [11.4, 16.5]			14.3 [11.7, 16.6]			14.0 [11.1, 16.4]			NS
Duration of follow-up	10.2 [5.7, 15.4]			11.3 [7.0, 15.5]			9.4 [4.5, 15.3]			NS
Laboratory variables at LN diagnosis										
Haemoglobin, g/dl	10.2	± 1.7		9.4	± 1.9		10.4	± 1.6		**0.045**
Leukocyte, × 10^9^/l	4.6 [3.2, 6.3]			5.1 [3.9, 7.3]			4.5 [3.1, 6.2]			NS
Platelets, × 10^9^/l	184 [99, 270]			156 [88, 206]			188 [111.5, 286.5]			NS
Serum creatinine, μmol/l	55.5 [45.3, 78.8]			64 [55, 112]			54 [45, 76]			**0.05**
eGFR, ml/min/1.73 m^2^	95.5	± 40.6		77.5	± 42.9		100.8	± 38.6		**0.026**
Spot urine Protein/Creatinine Ratio, mg/mg	2.5 [1.1, 5.5]			3.0 [1.6, 4.9]			2.4 [1.0, 5.5]			NS
Anti-dsDNA, IU/ml	232.6 ± 157.5			291.2 ± 249.8			220.7 ± 130.9			NS
C3, g/L	0.37 [0.25, 0.48]			0.33 [0.23, 0.50]			0.37 [0.27, 0.48]			NS
SLEDAI score	17 [12, 20]			16 [13, 24]			17 [12, 20]			NS
Major organ involvement^#^										
Neuropsychiatric manifestation	9		(10.7)	4		(21.1)	5		(7.7)	NS
Severe gastrointestinal manifestation	3		(3.6)	0		(0)	3		(4.6)	NS
Severe cardiovascular manifestation	1		(1.2)	0		(0)	1		(1.5)	NS
Additional antibodies profiles*										
Anti-Ro seropositivity	41	(51.3)		11	(64.7)		30	(47.6)		NS
Anti-LA seropositivity	7	(8.8)		1	(5.9)		6	(9.5)		NS
Anti-Sm seropositivity	18	(22.5)		6	(35.3)		12	(19.0)		NS
Anti-RNP seropositivity	23	(28.8)		6	(35.3)		17	(27.0)		NS
Anti-cardiolipin IgG/IgM seropositivity	22	(26.8)		5	(27.8)		17	(26.6)		NS
Lupus anticoagulant seropositivity	11	(13.3)		3	(16.7)		8	(12.3)		NS
Anti-nuclear cytoplasmic antibody (ANCA)	5	(7.5)		0			5	(9.6)		NS

Values are expressed as count (%), mean ± SD or median [IQR]

Severe gastrointestinal manifestation includes mesenteric vasculitis, lupus-associated pancreatitis and protein-losing enteropathy

Severe cardiovascular manifestation include myocarditis, excluding serositis/ pericardial effusion

*NS* = Not statistically significant, *p* value > 0.05

*SLE* systemic lupus erythematosus, *LN* lupus nephritis, *eGFR* estimated glomerular filtration rate, *SLEDAI* score, Systemic Lupus Erythematosus Disease Activity Index 2000, Anti-RNP, anti-ribonuclear protein RNP

^#^Neuropsychiatric manifestation is defined by the collection of clinical symptoms including aseptic meningitis, cerebrovascular disease, seizure, acute confusion, psychosis, with evidence from neuroimaging and/or electroencephalogram

*With missing data

### Histological findings and clinical presentations

**Fig. 2:**
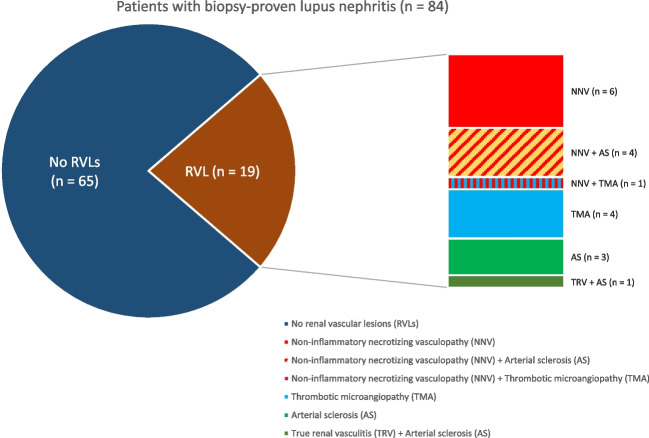
Composition of patients with biopsy-proven lupus nephritis in our cohort (*n* = 84)

Among the 19 patients with RVLs on kidney biopsy, six (31.6%) had isolated NNV, four (21.1%) had TMA and three (15.8%) had AS. Six patients had concurrent RVLs. Four patients (21.1%) had concurrent NNV and arterial sclerosis while one (5.3%) had both NNV and TMA in the same biopsy. The remaining one patient (5.3%) had both TRV and AS (Fig. 2). The clinical presentations of LN episodes at the time of kidney biopsies are shown in Table 2. Of the 107 LN episodes with biopsies performed, immunosuppression was prescribed before and after biopsy in 90 and 17 episodes, respectively. The rates of detecting RVLs were not statistically different between the two groups (18% vs.12%, *p* value = 0.73). LN episodes with histological evidence of RVLs showed more severe kidney manifestations, with higher serum creatinine (76 [Interquartile range IQR 55–174] vs.57 [IQR 46–79]μmol/l, *p* = 0.012), lower eGFR (66.9 ± 40.3 vs. 95.6 ± 39.4 ml/min/1.73 m^2^, p = 0.005) and higher risk of nephritic syndrome or stage 3 acute kidney injury (AKI) necessitating acute dialysis (57.9% vs.35.2%, *p* = 0.135). Haemoglobin (9.1 ± 1.9 vs.10.4 ± 1.9 g/dL, *p* = 0.008) and platelet levels (150.1 ± 96.4 vs.217.2 ± 104.8 × 10^9^/L, *p* = 0.012) were also lower in LN episodes with RVLs. Of note, the histological classification, activity index and chronicity scores were similar between LN episodes with or without RVLs (Table 3). We further analyzed the presence of crescents and sclerosed glomerulus by both percentage and scorings in the activity/ chronicity index, and there was no statistical difference (Supplementary Table 1). Histological findings were also not different between patients with NNV and those without RVL (Supplementary Table 2). Electron microscopy features are compared in Supplementary Table 3.

**Table 2:** Clinical characteristics among 107 biopsy episodes performed at initial presentation or kidney relapse

Clinical Characteristics	All (*n* = 107)		Episodes with RVL (*n* = 19)		Episodes without RVL (*n* = 88)		*P* value
Patient demographics							
Sex, Female	90	(84.1)	16	(84.2)	74	(84.1)	NS
Age of that episode, year	15.2 [12.1, 17.0]		15.9 [12.1, 17.4]		15.0 [12.1, 17.0]		NS
Episode							NS
Initial	83	(77.6)	14	(73.7)	68	(77.3)	
Relapse	24	(22.4)	5	(26.3)	20	(22.7)	
Clinical presentation							NS
Proteinuria	26	(24.3)	2	(10.5)	24	(27.3)	
Nephrotic syndrome	39	(36.4)	6	(31.6)	33	(37.5)	
Nephritic syndrome/ AKI requiring KRT	42	(39.3)	11	(57.9)	31	(35.2)	
Immunosuppressants before biopsy							
Yes	90	(84)	17	(89.5)	73	(83.0)	NS
Initial episode	62	(57)	12	(63.2)	50	(56.8)	NS
Duration from biopsy (days)	10	(7, 32)	10	(6, 30)	12	(6, 32)	NS
Relapse episode	25	(23.4)	5	(26.3)	23	(26.1)	
No	17	(15.9)	2	(10.5)	15	(17.0)	
Laboratory variables at episode presentation							
Haemoglobin, g/dl	10.2	± 1.9	9.1	± 1.9	10.4	± 1.9	**0.008**
Leukocyte, × 10^9^/l	5 [3.8, 7.1]		5.2 [3.3, 7.2]		5 [3.8, 7.1]		NS
Platelets, × 10^9^/l	205.3	± 106.1	150.1	± 96.4	217.2	± 104.8	**0.012**
Serum creatinine, μmol/l	60 [48, 95]		76 [55, 174]		57.0 [46, 79]		**0.012**
eGFR, ml/min/1.73 m^2^	90.5	± 40.9	66.9	± 40.3	95.6	± 39.4	**0.005**
Spot urine Protein/Creatinine Ratio, mg/mg	3.1 [1.4, 6.2]		3.5 [1.7, 6.3]		2.9 [1.2, 6.0]		NS
Anti-dsDNA, IU/ml	209.5	± 126.8	208.5	± 132.8	209.6	± 126.6	NS
C3, g/L	0.38 [0.29, 0.52]		0.35 [0.27, 0.50]		0.39 [0.30, 0.53]		NS
SLEDAI score	16 [12, 20]		16 [12, 22]		15.5 [12, 20]		NS

Values are expressed as count (%), mean ± SD or median [IQR]

*NS* = Not statistically significant, *p* value > 0.05

*AKI*, acute kidney injury, *KRT* kidney replacement therapy

**Table 3:** Histopathological features in biopsy-based episodes with and without RVLs

Histopathology	All (*n* = 107)		Episodes with RVL (*n* = 19)		Episodes without RVL (*n* = 88)		*P* value
ISN/RPS 2003 Class							NS
Class II	1	(0.9)	0		1	(1.1)	
Class III	22	(20.6)	6	(31.6)	19	(20.7)	
Class IV	43	(40.2)	7	(36.8)	36	(39.1)	
Class V	7	(6.5)	1	(5.3)	6	(6.5)	
Class III/IV + V	34	(31.8)	5	(26.3)	30	(32.6)	
Activity Index score	7.7	± 4.2	8.6	± 3.9	7.4	± 4.2	NS
Chronicity Index score	1.5	± 2.0	1.5	± 1.9	1.5	± 2.0	NS
Total score	9.2	± 4.6	10.1	± 4.5	9.0	± 4.7	NS

Values are expressed as count (%) or mean ± SD

*NS* = Not statistically significant, *p* value > 0.05

### Therapy

A significantly higher proportion of patients with RVLs received CYC-MMF as initial-maintenance therapy (52.6% vs.15.9%, *p* = 0.002), and therapeutic plasmapheresis (26.3% vs.1.1%, *p* ≤ 0.001) compared to those without RVLs (Table 4). The main indication for plasma exchange is TMA. The use of rituximab was similar between the two groups.

**Table 4:** Treatment regimen and treatment response in biopsy-based episodes with and without RVLs

	All **(***n* **= 107)**		Episodes with RVL (*n* = 19)		Episodes without RVL (*n* = 88)		*P* value
Treatment							
Corticosteroid	107	(100)	19	(100)	88	(100)	−
IV Methylprednisolone	61	(57.0)	14	(73.7)	47	(53.4)	NS
Initial-maintenance therapy							**0.002**
CYC-MMF	24	(22.4)	10	(52.6)	14	(15.9)	
MMF-MMF	40	(37.4)	5	(26.3)	35	(39.8)	
CYC-AZA	13	(12.1)	1	(5.3)	12	(13.6)	
AZA-AZA	11	(10.3)	0		11	(12.5)	
Triple Therapy	10	(9.3)	1	(5.3)	9	(10.2)	
Others	9	(8.4)	2	(10.5)	7	(8.0)	
Adjunctive treatment							
Plasmapheresis	6	(5.6)	5	(26.3)	1	(1.1)	** < 0.001**
Rituximab	10	(9.3)	3	(15.8)	7	(8.0)	NS
IVIg	2	(1.9)	2	(10.5)	0		**0.03**
Treatment response (KDIGO guideline)							
Response at 6 months							NS
Complete response	62	(57.9)	12	(63.2)	50	(56.8)	
Partial response	29	(27.1)	4	(21.1)	25	(28.4)	
No kidney response	16	(15.0)	3	(15.8)	13	(14.8)	
Response at 12 months							NS
Complete response	80	(74.8)	15	(78.9)	69	(73.9)	
Partial response	14	(13.1)	2	(10.5)	12	(13.6)	
No kidney response	13	(12.1)	2	(10.5)	11	(12.5)	

Values are expressed as count (%)

*NS* = Not statistically significant, *p* value > 0.05

Triple therapy included use of CNI in addition to corticosteroid and MMF. Other therapy included corticosteroid onotherapy, CYC-CYC, AZA-CNI, CNI-CNI

*AZA* azathioprine, *CNI* calcineurin inhibitors, *CYC* cyclophosphamide, *MMF* mycophenolate mofetil, *IVIg* intravenous immunoglobulin

### Short- and long-term kidney outcomes

The complete, partial, and non-remission rates at 6 and 12 months were comparable between the two groups (Table 5). Patients with and without RVLs also had similar kidney relapse (RVL group 6.5 episodes per 100 patient-year vs. non-RVL group 7.4 episodes per 100 patient-year, *p* = 0.69) (Table 5). At the last follow-up, the RVLs group had lower eGFR than the non-RVL group (95.1 ± 44.7 vs.114.1 ± 30.0 ml/min/1.73 m^2^, *p* = 0.046). There was no significant difference between both groups for serum creatinine and UPCR (Table 5). Subgroup analysis further characterised RVLs into NNV and other forms of RVLs (non-NNV group). Patients with NNV, compared to non-NNV and non-RVL groups, had a significantly higher serum creatinine at last follow-up (102 [IQR 58–220] vs. 62.5 [IQR 55–75] vs. 55 [IQR 48–64.5] μmol/l, p = 0.044). The NNV group also had a lower eGFR (79.6 ± 52.2 vs.116.3 ± 18.6 vs.114.1 ± 30.0 ml/min/1.73 m^2^, *p* = 0.089) despite not reaching statistical significance. No clinically significant difference in proteinuria was observed in all three groups of patients.

**Table 5:** Clinical outcomes of lupus nephritis with and without RVLs following treatment at last follow-up

	All *(n* = 84)		Patients with RVL (*n* = 19)		Patients without RVL (*n* = 65)	*p*-value
		All RVL (*n* = 19)	NNV (*n* = 11)	Non-NNV RVL (*n* = 8)		
Relapses						NS
No relapse	47 (56)	9 (47.4)	4 (36.4)	5 (62.5)	38 (58.5)	
One episode of relapse	23 (27.4)	7 (36.8)	5 (45.5)	2 (25)	16 (24.6)	
> 1 episode of relapse	14 (16.7)	3 (15.8)	2 (18.2)	1 (12.5)	11 (16.9)	
Development of CKD						NS
No CKD	71 (84.5)	14 (73.7)	7 (63.6)	7 (87.5)	57 (87.7)	
Stage 2	5 (6.0)	1 (5.3)	0	1 (12.5)	4 (6.2)	
Stage 3	1 (1.2)	0	0	0	1 (1.5)	
Stage 4	4 (4.8)	2 (10.5)	2 (18.2)	0	2 (3.1)	
Stage 5D	3 (3.6)	2 (10.5)	2 (18.2)	0	1 (1.5)	
Death	1 (1.2)	1 (5.3)	1 (9.1)	0	0	NS
Kidney parameters of last FU						
Serum creatinine, μmol/L	56 [49, 72]	64 [55, 93]	102 [58, 220]	62.5 [55, 75]	55 [48, 64.5]	**0.044**
eGFR, ml/min per 1.73 m^2^	109.8 ± 34.5	95.1 ± 44.7	79.6 ± 52.2	116.3 ± 18.6	114.1 ± 30.0	NS
Urine protein-to-creatinine ratio, mg/mg	0.11 [0.07, 0.24]	0.11 [0.06, 0.3]	0.11 [0.06, 0.98]	0.09 [0.06, 0.12]	0.1 [0.07, 0.24]	NS

Values are expressed as count (%), mean ± SD or median [IQR]

*P* value is calculated by comparing NNV, Non-NNV RVL and patients without RVL

*NS* = Not statistically significant, *p* value > 0.05

**Fig. 3:**
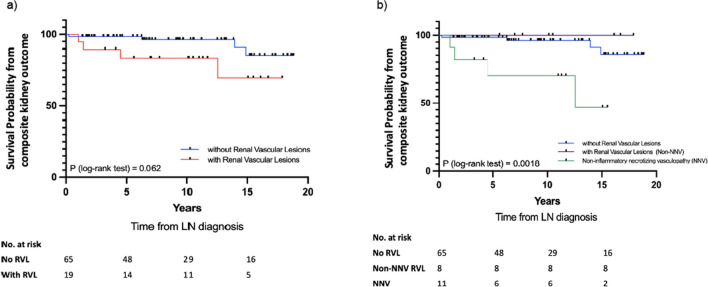
Kaplan–Meier plot of the composite outcome including advanced chronic kidney disease (stage 3–5), kidney failure and deaths in patients a) with and without RVLs; b) without RVLs, with Non-NNV RVLs and with NNV

The RVL group was associated with an inferior survival free from composite kidney outcomes, although it did not reach statistical significance (94.7% vs. 98.5%, 83.5% vs. 98.5%, 83.5% vs. 96.3% and 69.6% vs. 85.6% at 1, 5, 10 and 15 years, respectively; p = 0.062 by log-rank test) (Fig. 3a). The NNV group showed a significantly worse survival free from composite kidney outcomes when compared to the non-NNV and non-RVL groups (log-rank *p* = 0.0018; Fig. 3b). The 1-, 5-, 10- and 15-year survival free from composite kidney outcomes for NNV groups were 90.9%, 70.1%, 70.1% and 46.8%, respectively.

Univariate analysis showed that the presence of NNV (HR 7.08, 95% CI 1.67 – 30.03), along with acute dialysis at presentation (HR 10.18, 95% CI 1.70 – 61.10), higher chronicity score at biopsy (HR 1.76, 95% CI 1.14 – 2.73) and non-remission at 12 months after treatment (HR 8.58, 95% CI 2.00 – 36.86) were associated with adverse kidney outcome (Table 6).

**Table 6:** Univariate analysis of risk factor for progression to composite outcome of advanced CKD (stage 3–5), kidney failure and/or death

	Hazard ratio (95% CI)	*P* value
Age of SLE diagnosis, y	1.08 (0.84—1.38)	NS
Age of LN diagnosis, y	1.20 (0.93—1.55)	NS
Female*	−	−
eGFR at time of diagnosis	0.99 (0.97—1.01)	NS
Acute dialysis at presentation	10.18 (1.70—61.10)	**0.011**
SLEDAI (≥ 12 points)	1.34 (0.16—10.89)	NS
Number of kidney relapse	1.52 (0.87—2.63)	NS
Proliferative GN (III, IV, III/IV + V)*	−	−
Activity index score in 1st biopsy	1.08 (0.87—1.33)	NS
Chronicity index score in 1st biopsy	1.76 (1.14—2.73)	**0.011**
Renal vascular lesions		
No renal vascular lesions	1 [ref.]	−
All RVL	3.45 (0.86 – 13.80)	NS
Non-inflammatory necrotizing vasculopathy (NNV)	7.08 (1.67—30.03)	**0.005**
RVL other than NNV*	−	−
Remission status at/ before 12 months		
Complete/ Partial remission	1 [ref.]	−
Non-response	8.58 (2.00—36.86)	**0.004**

^*^ Hazard ratios are degenerated because either all or no events are present in the group

## Discussion

This is the first cohort study that reported the clinical epidemiology and significance of RVLs in cLN. In our study, RVLs were observed in 22.6% of patients with cLN. Importantly, the presence of RVLs, in particular, NNV, was associated with more severe clinical presentations and worse long-term kidney outcomes. This highlights the importance of including RVL as a part of the routine assessments on kidney histology in cLN.

Our results indicate that more than one-fifth of cLN patients showed RVLs on kidney biopsy. While cLN is often regarded as a more aggressive disease than adult-onset LN, the prevalence of RVLs in this study appeared to be lower than that in adults. This can be partly explained by the exclusion of ICD in our study. However, the prevalence of RVLs in adult-onset LN are highly variable (range between 28%—82%) and the types of RVLs reported were also rather heterogeneous [11, 19–22]. In our cohort, NNV and TMA were the most common RVLs on kidney biopsy in cLN. All currently available data on the types of RVLs in LN came from adult populations. While Wu et al. reported ICD represented 74.2% of RVLs, in other studies AS was observed in 24–57.8% of biopsies [11, 19–23]. The highly varying prevalence of RVLs could be a result of different classification definitions, patient populations, disease severity and duration, and evaluation methods.

NNV is distinguished from TRV by the absence of inflammatory cells in the blood vessel wall, and was the predominant RVL in our cohort, accounting for up to 57.9% of the RVLs. In adults, there is a considerable variation in its prevalence, ranging from 1.4 to 24% [11, 19–22]. RVL may exist as an isolated vascular lesion, though coexistence with other RVLs is not uncommon [24]. Its pathogenesis remains unclear. Nevertheless, with the presence of abundant immune complex deposits consisting of immunoglobulin and complements in the vessel walls, some postulate that immunologic factors play the main role in the evolution of NNV. It is also commonly associated with diffuse proliferative LN [10, 11, 19]. In addition, there may be extra-kidney involvements such as cardiac, neurological, and retinal vasculopathy [25–27]. In our cohort, four of our patients with NNV lesions developed neuropsychiatric lupus where the diagnosis was evidenced by neuroimaging. The presentations were heterogenous and significant, including vertical gaze palsy due to pathogenic lesion at the vertical gaze center [28]. Indeed, the presence of NNV on kidney biopsy is clinically important, as one-third of our patients with NNV reached the composite kidney outcome. Although half of these patients had co-existing AS, we believe these were likely to be the sequelae of the endothelial damage in necrotizing vasculopathy. Our data demonstrated that NNV was associated with worse long-term kidney outcome. In the adult population, there is conflicting evidence over the impact of NNV on long-term kidney outcome. Banfi et al. reported a lower 5-year kidney survival in patients with NNV (68.1 ± 10.2% vs. 89.6 ± 2.7%), compared to those without [19]. In contrast, Chu et al. and Mejia et al. did not observe such difference in kidney survival [21, 24].

Another important form of RVL is TMA, which accounted for 26.3% of the RVLs in our patients. In LN, its prevalence is reported to be between 0.5 to 24% [11, 19, 21, 22, 29–31]. The causes of TMA in the setting of LN include anti-phospholipid syndrome (APLS), thrombotic thrombocytopenic purpura (TTP) and complement-mediated TMA. TMA could also be associated with infection, malignant hypertension, drugs and other predisposing factors [17, 32]. In our cohort, two out of the five patients with TMA had a clinical diagnosis of TTP, although ADMAMTS13 tests were not available locally at the time of presentation. One patient was confirmed to have APLS. Prompt identification of these entities is important, where specific managements such as therapeutic plasma exchange or eculizumab may be instituted [17]. It is well recognized that adult LN patients with TMA often have severe AKI [29, 30, 33], and have worse long-term kidney prognosis [11, 30, 33]. Strufaldi et al. reported that patients with TMA had a significantly lower eGFR at biopsy and were more likely to develop kidney failure and mortality (82.9% vs.32.9%) [33]. Li et al. also reported a lower 5-year kidney survival (70% vs. 95%) in patients with LN and TMA [30]. In contrast, while 60% of our subjects with TMA necessitated acute dialysis support, their kidney function improved and none of them developed composite kidney outcome at last follow-up. AS was observed in 42% of RVL in our cohort but the majority of them appeared concurrently with other forms of RVL. This suggests that AS may be a sequela following active inflammation of the blood vessels, rather than the primary pathological event.

In our study, patients with RVLs had severe presentation with more severe kidney impairment and haematological manifestations when compared to the non-RVLs group. Our results also concurred with previous studies which demonstrated a significantly higher proportion of patients required acute dialysis support [11, 21]. Importantly, both LN classification and activity index were not different between the two groups, and hence suggests the presence of RVLs on kidney biopsy portends more severe clinical manifestations. Despite a satisfactory and comparable treatment response and remission status at 6 and 12 months in both groups, our results showed that patients with RVL tended to have worse long-term kidney outcome. The failure to reach statistical significance might be explained by small patient number in the RVL group. Another possible explanation is that more aggressive immunosuppressive therapy, such as cyclophosphamide, was offered to the RVL group, which led to similar disease remission rates in the short term. Notwithstanding, the presence of NNV was an independent predictor of adverse kidney outcome according to our data. Lastly, whereas kidney relapse occurs in 50% of patients in 10 years’ time [34], our data did not support a significant association between RVLs and the development of kidney relapse. The current study thus suggests a potential relationship between the presence of RVL and worse kidney outcomes. Future study would be required to demonstrate whether RVL is a strong independent predictor on long-term outcomes. We believe our data highlight the importance of evaluating these histological lesions regularly, and their incorporation into the current LN activity/ chronicity scoring system should be considered to better assess disease severity and prognosis.

In our cohort, most patients were receiving immunosuppression at the time of kidney biopsy. There were several reasons for this practice. First, being the territory-wide paediatric nephrology referral centre in Hong Kong, a large proportion of our patients were referred from other general paediatric units where kidney biopsy service was not readily available. Prior to transfer, these patients might have been treated as having LN based on clinical and serological markers. Second, a subgroup of patients had severe organ- and life-threatening presentations which warranted immediate immunosuppressive therapies. Lastly, since kidney biopsy was performed by interventional radiologists in our hospital, there was a short waiting time to scheduled appointment. While the timing and proportion of patients receiving immunosuppression prior to kidney biopsy were comparable between the RVL and non-RVL groups, the use of immunosuppressants could potentially influence the histological findings.

Our study has a few limitations. First, due to limited patient numbers, we were unable to analyse each sub-type of RVLs individually. Around one-third of our cohort also had concurrent RVLs, which may confound the long-term outcomes. Second, excluding ICD in our cohort underestimates the prevalence of RVL in cLN though a previous report saw it as a hallmark of LN instead of a lesion by itself [20]. Third, while kidney biopsies were routinely performed at initial presentation, the indication for repeating biopsies remained controversial and were individualized according to disease severity. Fourth, management of RVL was non-specific, and depended on both clinical severity and histological classifications, which might have confounded short- and long-term outcomes. Finally, the study is limited by its retrospective nature with potential referral and reporting bias. Nevertheless, our study has a relatively large patient number with long follow-up period and minimal missing data.

In conclusion, RVL is not uncommon and can occur in one-fifth of children and adolescents with LN. The presence of these lesions, particularly NNV, is associated with severe disease presentation and worse long-term kidney outcomes. Our data supports the incorporation of RVLs into the NIH activity and chronicity indices for better prediction of long-term outcomes. This may potentially help individualization of treatments to mitigate the need for acute dialysis and achieve satisfactory disease control [35].

## Supplementary Information

Below is the link to the electronic supplementary material.

Graphical abstract (PPTX 288 KB)

Supplementary file2 (DOCX 19 KB)

## Data Availability

The dataset generated during and/or analysed during the current study are available from the corresponding author on reasonable request.
