# Supplementary material for: Renal vascular lesions in childhood-onset lupus nephritis
Source: Pediatr Nephrol. 2024 Sep 9;40(1):131–41. doi: 10.1007/s00467-024-06498-z (PMC11584461; doi:10.1007/s00467-024-06498-z)
Supplement: Supplementary file 1 — Graphical abstract (PPTX 288 KB) [file 467_2024_6498_MOESM1_ESM.pptx]

## Slide 1
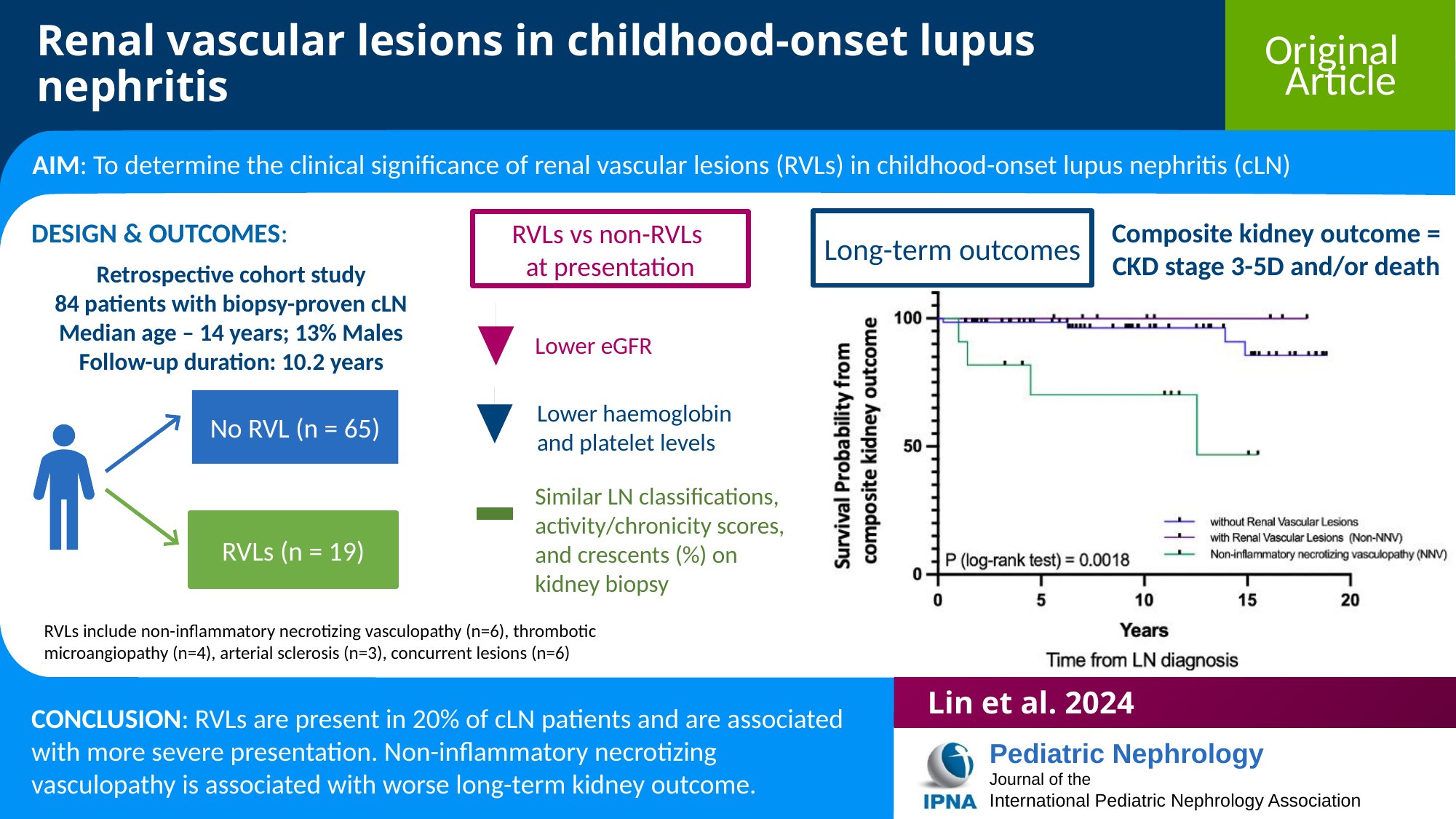

Renal vascular lesions in childhood-onset lupus nephritis
AIM: To determine the clinical significance of renal vascular lesions (RVLs) in childhood-onset lupus nephritis (cLN)
Composite kidney outcome = CKD stage 3-5D and/or death
DESIGN & OUTCOMES:
Long-term outcomes
RVLs vs non-RVLs
at presentation
Retrospective cohort study
84 patients with biopsy-proven cLN
Median age – 14 years; 13% Males
Follow-up duration: 10.2 years
Lower eGFR
No RVL (n = 65)
Lower haemoglobin and platelet levels
Similar LN classifications, activity/chronicity scores, and crescents (%) on kidney biopsy
RVLs (n = 19)
RVLs include non-inflammatory necrotizing vasculopathy (n=6), thrombotic microangiopathy (n=4), arterial sclerosis (n=3), concurrent lesions (n=6)
Lin et al. 2024
CONCLUSION: RVLs are present in 20% of cLN patients and are associated with more severe presentation. Non-inflammatory necrotizing vasculopathy is associated with worse long-term kidney outcome.
