# Supplementary material for: Renal vascular lesions in childhood-onset lupus nephritis
Source: Pediatr Nephrol. 2024 Sep 9;40(1):131–41. doi: 10.1007/s00467-024-06498-z (PMC11584461; doi:10.1007/s00467-024-06498-z)
Supplement: Supplementary file 2 — Supplementary file2 (DOCX 19 KB) [file 467_2024_6498_MOESM2_ESM.docx]

Supplementary Table 1

|  | **Episodes with RVL**  **(n= 19)** | **Episodes without RVL**  **(n = 88)** | **P value** |
| --- | --- | --- | --- |
| Cellular/ Fibrocellular crescent% (median) | 0 [0, 15] | 0 [0, 6] | 0.18 |
| Cellular/ Fibrocellular crescent in activity index (median) | 0 [0, 2] | 0 [0, 2] | 0.25 |
| Sclerosed glomerular% (median) | 0 [0, 0] | 0 [0, 0] | 0.20 |
| Total glomerulosclerosis score in chronicity index (median) | 0 [0, 0] | 0 [0, 0] | 0.25 |

Values are expressed as median [IQR]

Supplementary Table 2

| **Histopathology** | **All (n = 107)** | | **Episodes with NNV**  **(n = 11)** | **Episodes without RVL (n = 88)** | **P value** |
| --- | --- | --- | --- | --- | --- |
| ISN/RPS 2003 Class |  |  |  |  |  |
| Class II | 1 | (0.9) | 0 | 1 (1.1) | NS |
| Class III | 22 | (20.6) | 3 (27.3) | 19 (20.7) |  |
| Class IV | 43 | (40.2) | 4 (36.4) | 36 (39.1) |  |
| Class V | 7 | (6.5) | 0 | 6 (6.5) |  |
| Class III/IV + V | 34 | (31.8) | 3 (27.3) | 30 (32.6) |  |
| Activity Index score | 7.7 | ± 4.2 | 9.1 ± 3.5 | 7.4 ± 4.2 | 0.11 |
| Chronicity Index score | 1.5 | ± 2.0 | 1.6 ± 2.2 | 1.5 ± 2.0 | 0.43 |
| Total score | 9.2 | ± 4.6 | 10.7 ± 4.8 | 9.0 ± 4.7 | 0.12 |
| Values are expressed as counts (%) or mean ± SD  NS = Not statistically significant, p value > 0.05 | | | | | |

Supplementary Table 3

| **Electron Microscopy (EM) features** | **Episodes with RVL (n=19)**  **EM available (n=17)** | **Episodes without RVL (n=88)**  **EM available (n=72)** | **P value** |
| --- | --- | --- | --- |
| Immune complex deposit |  |  |  |
| Predominantly subendothelial | 11 (64.7) | 38 (52.8) | 0.83 |
| Small amount (<50%) | 5 (29.4) | 15 (20.8) |  |
| Moderate to large (>=50%) | 6 (35.3) | 23 (31.9) |  |
| Predominantly subepithelial | 1 (5.9) | 6 (8.3) |  |
| Co-predominant subendothelial and subepithelial (>=50%) | 5 (29.4) | 28 (38.9) |  |
|  |  |  |  |
| Glomerular basement membrane |  |  |  |
| Normal thickness | 12 (70.6) | 54 (75) | 0.82 |
| Focal thickening (<50%) | 3 (17.6) | 11 (15.3) |  |
| Diffuse thickening (>=50%) | 2 (11.8) | 7 (9.7) |  |
|  |  |  |  |
| Foot process alteration |  |  |  |
| Focal (< 50%) | 5 (29.4) | 16 (22.2) | 0.30 |
| Diffuse (>= 50%) | 12 (70.6) | 56 (77.8) |  |
| Values are expressed as counts (%) | | | |
